# Prediction of knee biomechanics with different tibial component malrotations after total knee arthroplasty: conventional machine learning vs. deep learning

**DOI:** 10.3389/fbioe.2023.1255625

**Published:** 2024-01-08

**Authors:** Qida Zhang, Zhuhuan Li, Zhenxian Chen, Yinghu Peng, Zhongmin Jin, Ling Qin

**Affiliations:** ^1^ Musculoskeletal Research Laboratory, Department of Orthopaedics and Traumatology, The Chinese University of Hong Kong, Hong Kong, Hong Kong SAR, China; ^2^ State Key Laboratory for Manufacturing System Engineering, School of Mechanical Engineering, Xi’an Jiaotong University, Xi’an, China; ^3^ Key Laboratory of Road Construction Technology and Equipment (Ministry of Education), School of Mechanical Engineering, Chang’an University, Xi’an, China; ^4^ CAS Key Laboratory of Human-Machine Intelligence-Synergy Systems, Shenzhen Institutes of Advanced Technology Chinese Academy of Sciences, Shenzhen, China; ^5^ Tribology Research Institute, School of Mechanical Engineering, Southwest Jiaotong University, Chengdu, China; ^6^ Institute of Medical and Biological Engineering, School of Mechanical Engineering, University of Leeds, Leeds, United Kingdom

**Keywords:** total knee arthroplasty, accurate rotational alignment, musculoskeletal multibody dynamics model, deep learning, machine learning, biomechanics

## Abstract

The precise alignment of tibiofemoral components in total knee arthroplasty is a crucial factor in enhancing the longevity and functionality of the knee. However, it is a substantial challenge to quickly predict the biomechanical response to malrotation of tibiofemoral components after total knee arthroplasty using musculoskeletal multibody dynamics models. The objective of the present study was to conduct a comparative analysis between a deep learning method and four conventional machine learning methods for predicting knee biomechanics with different tibial component malrotation during a walking gait after total knee arthroplasty. First, the knee contact forces and kinematics with different tibial component malrotation in the range of ±5° in the three directions of anterior/posterior slope, internal/external rotation, and varus/valgus rotation during a walking gait after total knee arthroplasty were calculated based on the developed musculoskeletal multibody dynamics model. Subsequently, deep learning and four conventional machine learning methods were developed using the above 343 sets of biomechanical data as the dataset. Finally, the results predicted by the deep learning method were compared to the results predicted by four conventional machine learning methods. The findings indicated that the deep learning method was more accurate than four conventional machine learning methods in predicting knee contact forces and kinematics with different tibial component malrotation during a walking gait after total knee arthroplasty. The deep learning method developed in this study enabled quickly determine the biomechanical response with different tibial component malrotation during a walking gait after total knee arthroplasty. The proposed method offered surgeons and surgical robots the ability to establish a calibration safety zone, which was essential for achieving precise alignment in both preoperative surgical planning and intraoperative robotic-assisted surgical navigation.

## 1 Introduction

Accurate alignment of tibiofemoral components is a critical element in obtaining favorable clinical outcomes for patients after total knee arthroplasty (TKA). Poor rotational alignment of tibiofemoral components can result in knee stiffness (Bedard et al., 2011; Kim et al., 2014), evaluated joint contact stress (Chen et al., 2015; Ueyama et al., 2020; Tang et al., 2022), and a high prevalence of TKA revisions (Dalury et al., 2013; Panni et al., 2018; Rajgopal et al., 2022). Moreover, over 50% of patients who experienced joint pain after TKA had mal-rotational alignment of the knee components, which is a substantial contributor to joint pain and functional deficit (Hofmann et al., 2003; Bell et al., 2014; Abdelnasser et al., 2019b; Rajgopal et al., 2022). The focus of most clinical studies has been on assessing the impact of component malrotation on knee function through postoperative evaluations. Notwithstanding, these assessments fail to provide surgeons accurately and quickly with the necessary biomechanical performance data for preoperative surgical planning or intraoperative surgical guidance. This lack of information may result in unsatisfactory recovery of patient knee function after TKA caused by component malrotation. Barrack et al. evaluated the relationship between anterior knee pain and component rotation after TKA, discovering that patients with anterior knee pain had an average of 6.2° of internal rotation compared to a mere 0.4° of external rotation in pain-free patients (Barrack et al., 2001). Similarly, Abdelnasser et al. investigated the effects of intra-operative intentional malrotation of the tibial component on vivo kinematics, revealing that internal rotation of the tibial component in TKA can result in postoperative extension deficits, potentially causing pain and knee stiffness (Abdelnasser et al., 2019a). These clinical findings underscored the importance of accurate component alignment in determining the knee function of TKA patients. Therefore, developing effective and quick methods to correct component malrotation during preoperative planning and intraoperative surgical guidance to prevent unsatisfactory functional recovery after surgery remains a pressing clinical challenge.

Numerous computational studies have explored the impact of component malrotation on knee biomechanics during a walking gait after TKA using musculoskeletal multibody dynamics and finite element methods (Kuriyama et al., 2014; Smith et al., 2016; Vanheule et al., 2017; Fang et al., 2022; Tang et al., 2022). Chen et al. found that varus-valgus malrotation of the tibial/femoral component and internal-external malrotation of the femoral component with a 5° variation impacted peak medial contact force by 17.8%–53.1%, peak lateral contact force by 35.0%–88.4%, and peak total contact force by 5.2%–18.7% using a multibody dynamics model (Chen et al., 2015). Likewise, Kang et al. demonstrated that external malrotation of the femoral component increased the lateral contact stress of the tibial component, whereas internal malrotation increased the medial contact stress using a finite element method (Kang et al., 2016). Nonetheless, these computational models have a significant limitation in that they are typically time-consuming and computationally demanding. As a result, accurately and quickly predicting the biomechanical response to component malrotation during preoperative surgical planning or intraoperative surgical guidance using computational models remains a considerable challenge.

Recently, artificial intelligence (AI) techniques have emerged as a promising alternative for quickly and accurately predicting human biomechanics. Numerous studies have employed machine learning methods to forecast ground reaction forces from patient gaits (Oh et al., 2013; Guo et al., 2017; Wouda et al., 2018; Johnson et al., 2019; Komaris et al., 2019), computer vision methods to estimate patient poses (Mehrizi et al., 2019; Tamura et al., 2020), and wearable sensors to assess patient kinematics (Stetter et al., 2019; Gholami et al., 2020; Mundt et al., 2020). Stetter et al. demonstrated that combining wearable sensors and artificial neural networks accurately estimated knee joint forces across various movements, including linear motions, changes of direction, and jumps (Stetter et al., 2019). Zhu et al. proposed a knee contact force prediction method that integrated artificial fish swarm and random forest algorithms, with experiments verified that the proposed method outperformed classical multibody dynamics analysis and artificial neural network models (Zhu et al., 2020). Moreover, Rane et al. trained a deep neural network using a set of kinematic, kinetic, and electromyographic measurements from 156 subjects during gaits and showed that the magnitudes of the medial knee joint force and muscle forces predicted by the proposed method were in good agreement with those derived from musculoskeletal modeling. (Rane et al., 2019). However, there is a paucity of literature on the development of machine/deep learning methods for accurately predicting knee biomechanics with different tibial component malrotation in TKA. Additionally, it remains uncertain whether a noteworthy distinction exists between the deep learning method and conventional machine learning methods in predicting knee biomechanics under different tibial component malrotations during a walking gait in TKA. Therefore, the development of machine/deep learning methods for accurate and rapid prediction of knee biomechanics with different tibial component malrotations during a walking gait in TKA is essential for preoperative surgical planning and intraoperative robotic-assisted surgical navigation.

The objective of the present study was to: 1) develop deep learning method and conventional machine learning methods to predict knee biomechanics with different tibial component malrotation during a walking gait after TKA using the dataset derived from the developed musculoskeletal multibody dynamics model; 2) conduct a comparative analysis between the deep learning method and conventional machine learning methods for predicting knee contact forces and kinematics with different tibial component malrotation during a walking gait after TKA.

## 2 Materials and methods

### 2.1 Musculoskeletal multibody dynamics model

**FIGURE 1:**
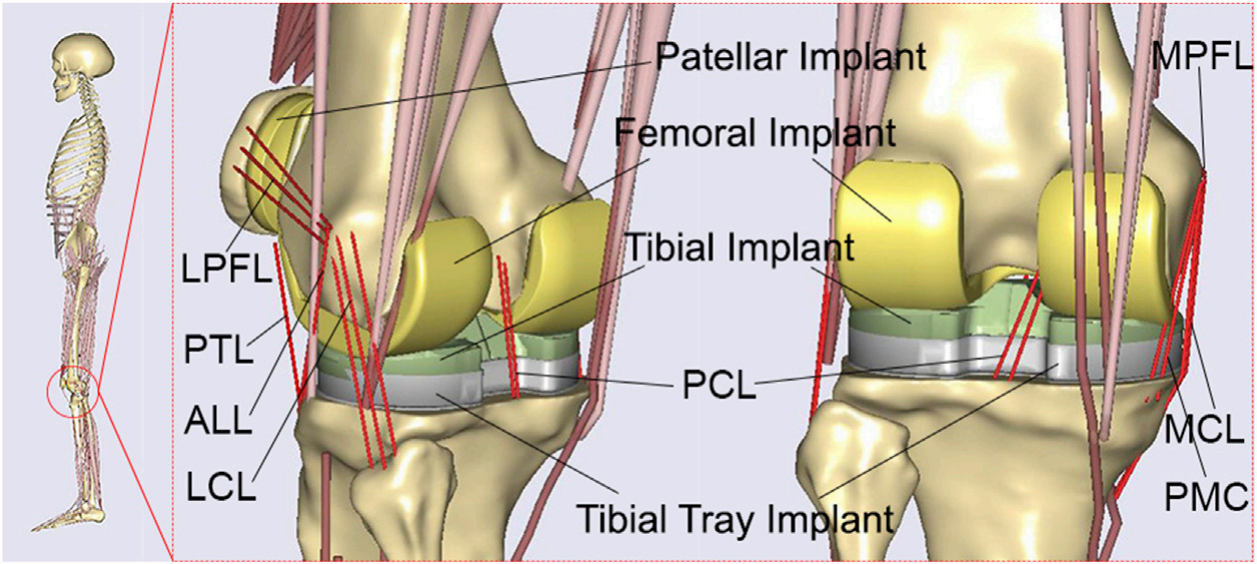
The developed patient-specific musculoskeletal multibody dynamics model of total knee arthroplasty.

A patient-specific musculoskeletal multibody dynamics model of TKA (Chen et al., 2016; Zhang et al., 2019; Zhang et al., 2020) was developed using Anybody modeling system (version 6.0; AnyBody Technology, Aalborg, Denmark), based on an advanced bone morphing technique utilizing the patient’s preoperative and postoperative computed tomography (CT) scans (Damsgaard et al., 2006; Pellikaan et al., 2014) (Figure 1). Data from a TKA patient (gender: male; mass: 75 kg; height: 180 cm; surgery: left knee), obtained from the publicly SimTK website (https://simtk.org/home/kneeloads), was employed to develop the patient-specific musculoskeletal model (Fregly et al., 2012). The patient’s database included the geometries of knee implants and the lower limb bones derived from patient CT scans. In addition, marker trajectories and ground reaction forces obtained from motion capture experiments were included in the database. The segments and each muscle’s isometric strength were scaled according to the patient’s weight and height utilizing a length-mass-fat scaling approach (Rasmussen et al., 2005; Lund et al., 2015; Marra et al., 2015; Chen et al., 2016; Hu et al., 2019). An innovative knee joint model consisting of 11 degrees of freedom was established via the force-dependent kinematics method, including deformable contact models of the artificial knee joint (Andersen and Rasmussen, 2011; Marra et al., 2015; Chen et al., 2016). The tibiofemoral joint had 6° of freedom, and the patellofemoral joint had 5° of freedom, assuming a rigid patellar tendon ligament (PTL) (Chen et al., 2016). Three deformable contact models were defined between the femoral component and the medial/lateral tibial components, as well as between the femoral component and the patellar component, based on the elastic foundation theory (Fregly et al., 2003). The knee joint model was enveloped by ligaments including the medial collateral ligament (MCL), lateral collateral ligament (LCL), posterior cruciate ligament (PCL), posterior-medial capsule (PMC), anterior-lateral ligament (ALL), medial patellofemoral ligament (MPFL), and lateral patellofemoral ligament (LPFL) (Chen et al., 2016; Zhang et al., 2020). These ligaments were modeled as nonlinear spring elements with a piecewise force-displacement relationship (Blankevoort et al., 1991). An inverse kinematics method (Andersen et al., 2010) was conducted to determine the pelvic motion, hip angles, and foot locations based on the walking gait data, the scaled musculoskeletal model, and the optimized marker locations (Marra et al., 2015; Chen et al., 2016). These kinematics and ground reaction forces were subsequently input into the inverse dynamics analysis, which incorporated the force-dependent kinematics method (Andersen and Rasmussen, 2011; Marra et al., 2015; Chen et al., 2016), to calculate tibiofemoral contact forces and kinematics. In the inverse dynamics analysis, a cubic polynomial muscle recruitment criterion was also adopted to determine which set of muscles will balance a given external load. Additional information regarding the development of the musculoskeletal multibody dynamics model of TKA can be found in our previous studies (Chen et al., 2016; Zhang et al., 2019; Zhang et al., 2020).

**FIGURE 2:**
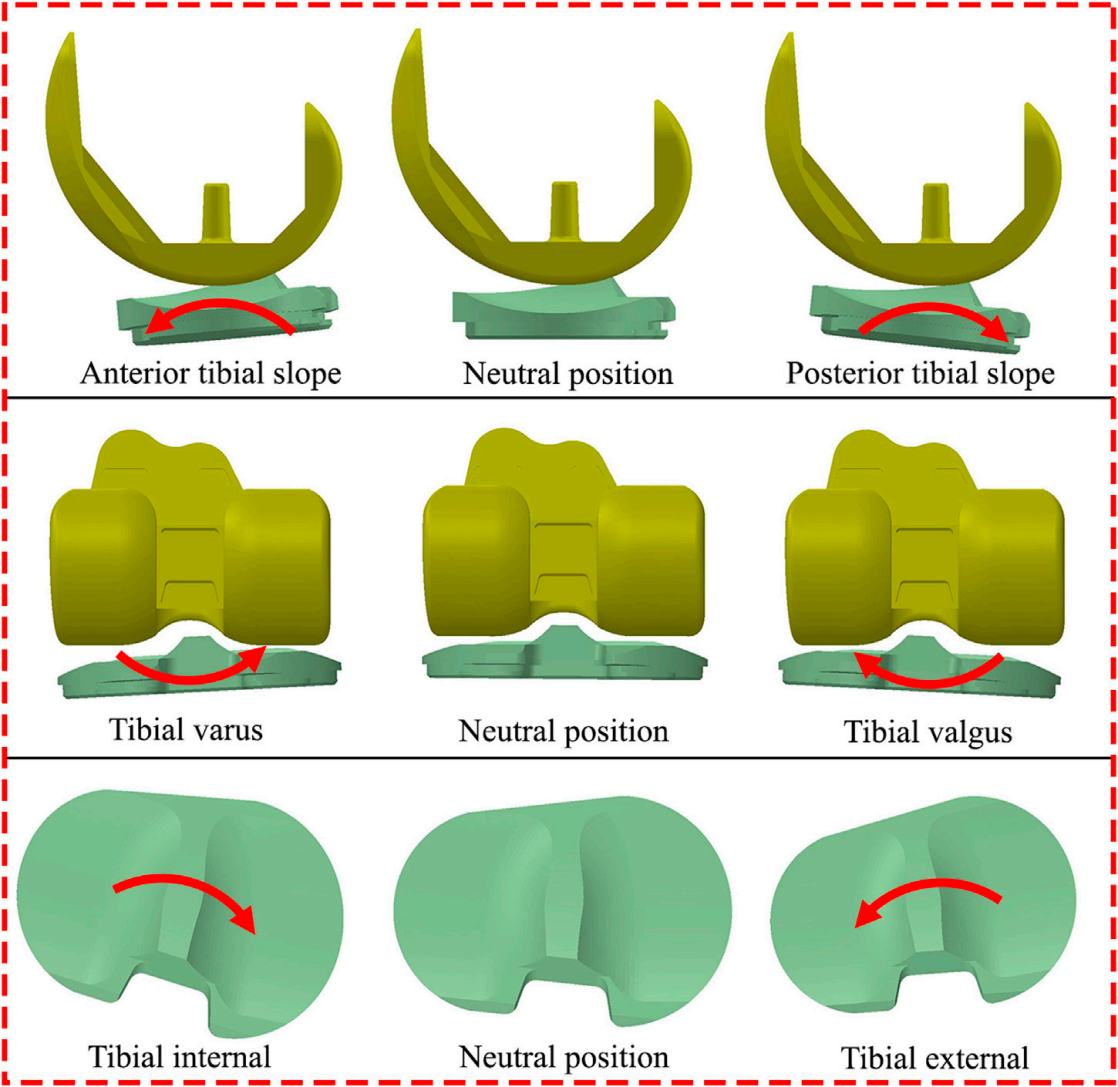
Schematic diagram for different tibial component malrotation.

As per the patient’s surgical report (Fregly et al., 2012; Chen et al., 2015; Chen et al., 2016), two 90° cuts were made on the proximal tibia in the coronal and sagittal planes with respect to the long axis. The distal femur was cut at 6° valgus from the anatomical axis. A 3° external rotation cut was made on the posterior femur with respect to the posterior condyles. These cuts were defined as the neutral position of the femoral and tibial components in the developed patient-specific musculoskeletal multibody dynamics model of TKA. To investigate the effect of tibial component malrotation, the rotational positions of the tibial component were modified from the neutral position in 343 cases: neutral, ±1°, ±3°, and ±5° of anterior-posterior slope, ±1°, ±3° and ±5° of internal-external rotation, and ±1°, ±3° and ±5° of varus-valgus rotation (Figure 2). Each tibial component with the same femoral component was separately imported into the developed patient-specific musculoskeletal multibody dynamics model of TKA. The knee contact forces and kinematics during a walking gait under different tibial component malrotation in the range of ±5° in the three directions of anterior/posterior slope, internal/external rotation, and varus/valgus rotation were calculated. These results will serve as a dataset to develop deep learning and machine learning methods (Table 1).

**TABLE 1:** Predictor and predicted features used in deep learning and machine learning methods.

Predictor features		Predicted features	
Tibial Component Malrotation	Anterior-Posterior Slope	Contact Forces	Total Contact Force
			Medial Contact Force
			Lateral Contact Force
	Internal-External Rotation		Flexion-Extension Rotation
		Kinematics	Internal-External Rotation
			Varus-Valgus Rotation
	Varus-Valgus Rotation		Anterior-Posterior Translation
			Proximal-Distal Translation
			Medial-Lateral Translation

### 2.2 Deep learning model

A deep learning model using recurrent neural networks (RNNs) was developed to predict knee contact forces and kinematics with different tibial component malrotations over a walking gait cycle. The model utilized predictor features of tibial component malrotation, including anterior-posterior slope, internal-external rotation, and varus-valgus rotation (Figure 2; Table 1). The predicted features included knee contact forces and kinematics (Table 1). The RNN architecture consisted of two bi-directional long short-term memory (LSTM) cells (Hochreiter and Schmidhuber, 1997) and three fully-connected layers. Different tibial component malrotation were represented by $x^{i}\in R^{A\times T}$ with *A* tibial component malrotation variables over T time steps. The knee biomechanics over a walking gait cycle were obtained using the musculoskeletal multibody dynamics model and stored in $y^{i}\in R^{B\times T}$ with B output variables. RNNs were trained to with the aim of minimizing the objective function:

$$J\left(X,Y,\theta \right)=\frac{1}{N}\;\sum_{i=1}^{N}{\left\|y^{i}-\;{\hat{y}}^{i}\right\|}^{2}+\lambda {\left\|\theta \right\|}^{2}$$

The training set, $X=\left\{x^{1},x^{2},\ldots ,x^{N}\right\}$ and $Y=\left\{y^{1},y^{2},\ldots ,y^{N}\right\}$ , comprised different tibial component malrotation and corresponding knee biomechanics derived from the musculoskeletal model. RNNs predicted knee biomechanics time series trends ( ${\hat{y}}^{i}$ ) based on the corresponding predictor features (different tibial component malrotation). Here, *λ* is the regularization parameter, which controls the trade-off between fitting the training data and keeping the parameter values small. *θ* is the parameter vector that the model aims to learn. It contains the weights associated with each feature in the input matrix *X*.

RNNs were trained for 10,000 iterations using Adam optimization with a learning rate of 0.0001 (Kingma and Ba, 2014). The weights of the RNNs were randomly initialized from a Gaussian distribution (μ = 0, *σ* = 0.01). An L2 regularization (*λ* = 0.001) and batch size of 2 were employed. These hyperparameters were fine-tuned based on our dataset and experimental setup, aiming to optimize the training process and enhance the model’s performance. PyTorch served as the implementation framework for the deep learning model in this study (Paszke et al., 2017).

### 2.3 Machine learning model

Similarly, four ensemble learning methods were developed to predict knee contact forces and kinematics with different component malrotation over a walking gait cycle. These models utilized predictor features of tibial component malrotation, including anterior-posterior slope, internal-external rotation, and varus-valgus rotation (Figure 2; Table 1). The predicted features included knee contact forces and kinematics (Table 1). Four ensemble learning methods were developed using Scikit-learn in Python (Pedregosa et al., 2012; Buitinck et al., 2013): Random Forest regression, AdaBoost regression, Gradient Boosting regression, Voting regression. Ensemble methods aimed to enhance the generalizability and robustness of a single estimator by combining the predictions of multiple base estimators built using a given learning algorithm (Pedregosa et al., 2012).

The Random Forest algorithm (Breiman, 2001; Pedregosa et al., 2012) was applied in this study, which involved merging *k* base learned models (*M*_*1*_, *M*_*2*_, …, *M*_*K*_) to construct an enhanced composite prediction model, denoted as M*. To generate base model *M*_*i*_, *k* training sets [*D*_*1*_, *D*_*2*_, …, *D*_*K*_, where *D*_*i*_ (1 ≤ *i ≤ k*)] were derived from a given dataset *D*. The given dataset in this study referred to the original dataset that contained the input features (e.g., tibial component malrotation features like anterior-posterior slope, internal-external rotation, varus-valgus rotation) and corresponding output variables (knee contact forces and kinematics). The given dataset was used to generate k training datasets through a technique called bootstrap aggregating or “bagging.” Bagging involved random sampling with replacement from the given dataset, resulting in k unique training datasets (D_1_, D_2_, …, D_K_). Each training dataset was used to train a separate base model (M_1_, M_2_, …, M_K_) within the Random Forest ensemble. The purpose of creating multiple training datasets was to introduce diversity among the base models, as each base model was trained on a different subset of the original dataset. When a new data tuple was presented to the ensemble model, each base model returned a predicted result, and the ensemble model produced the final prediction by averaging or taking the mode of the predicted results from the base models (Pedregosa et al., 2012; Zhu et al., 2020).

The AdaBoost algorithm (Freund and Schapire, 1997; Pedregosa et al., 2012) was employed in this study, utilizing sequential weak learners, which were models that only marginally outperform random guessing, such as small decision trees, to repeatedly modified versions of the data. These weak learners were iteratively applied to modified versions of the data. The final prediction was obtained by combining the predictions of all weak learners through a weighted majority vote or sum. During each boosting iteration, the weights w_1_, w_2_, …, w_N_ assigned to each training sample were adjusted. Initially, all weights were set to *w*_*i*_ = 1/*N*, enabling the first iteration to train a weak learner on the original data (Pedregosa et al., 2012). Subsequently, at each iteration, the weights of the training samples were individually modified, and the learning algorithm was reapplied to the reweighted data. Specifically, for a given iteration, the weights of training examples incorrectly predicted by the previous boosted model were increased, while the weights of correctly predicted examples were decreased (Pedregosa et al., 2012). As the iterations progressed, examples that were challenging to predict received progressively greater influence. This iterative adjustment of weights compelled each subsequent weak learner to focus on the examples that were previously missed by the preceding weak learners in the sequence (Drucker, 1997; Hastie et al., 2009; Pedregosa et al., 2012). By iteratively adjusting the weights and training weak learners, AdaBoost constructed a strong ensemble model that combines the predictions of the individual weak learners to improve overall regression prediction performance.

The Gradient Boosting algorithm, as proposed by Friedman and refined by Pedregosa et al., leveraged the combination of multiple weak learners to construct robust ensemble models (Friedman, 2001; Pedregosa et al., 2012). By employing gradient descent optimization, each subsequent model was trained to minimize the loss function, such as mean squared error, of the previous model. During each iteration, the algorithm calculated the gradient of the loss function with respect to the predictions of the current ensemble and utilized this gradient to train a new weak model. The objective was to minimize the gradient, thereby improved the overall performance of the ensemble. The predictions of the newly trained model were then integrated into the ensemble, reinforcing the collective predictive capability. This iterative process continued until a predefined stopping criterion was satisfied (Pedregosa et al., 2012).

The voting regression algorithm combined different machine learning regression algorithms and returned the average predicted values (Pedregosa et al., 2012). In this approach, multiple regression models were trained using various algorithms, including Random Forest, AdaBoost and Gradient Boosting. Each individual model generated its prediction for a given input, and the final prediction was obtained by averaging the predicted values of all models. The voting algorithm allowed for a more robust prediction by considering the collective knowledge of multiple models and balancing out their individual weaknesses. The voting regression approach was particularly useful when the individual models had similar performance levels, as it leveraged their combined strengths to improve overall prediction accuracy (Pedregosa et al., 2012).

### 2.4 Performance analyses

The deep learning and learning models developed in this study were evaluated for accuracy using 5-fold cross-validation, where the dataset was divided into 5 equal subgroups used for the training and validation (Burton et al., 2021). An additional test dataset withheld from the cross-validation was used to confirm the performance of the models. To ensure consistency across all deep learning and machine learning algorithms, the same cross-validation splits were used for all models. To evaluate the models, each subgroup was held out for evaluation once, and all models were trained five times. The performance of the developed deep learning and machine learning models was assessed against musculoskeletal multibody dynamics model outputs, which were considered the ground truth. The evaluation was conducted using two metrics: Root mean square error (RMSE) and Pearson correlation coefficient. The Pearson correlation coefficient *ρ* was classified as weak (*ρ* ≤ 0.35), moderate (0.35 < *ρ* ≤ 0.67), strong (0.67 < *ρ* ≤ 0.9), and excellent (0.9 < *ρ*) according to the obtained values.

## 3 Results

**FIGURE 3:**
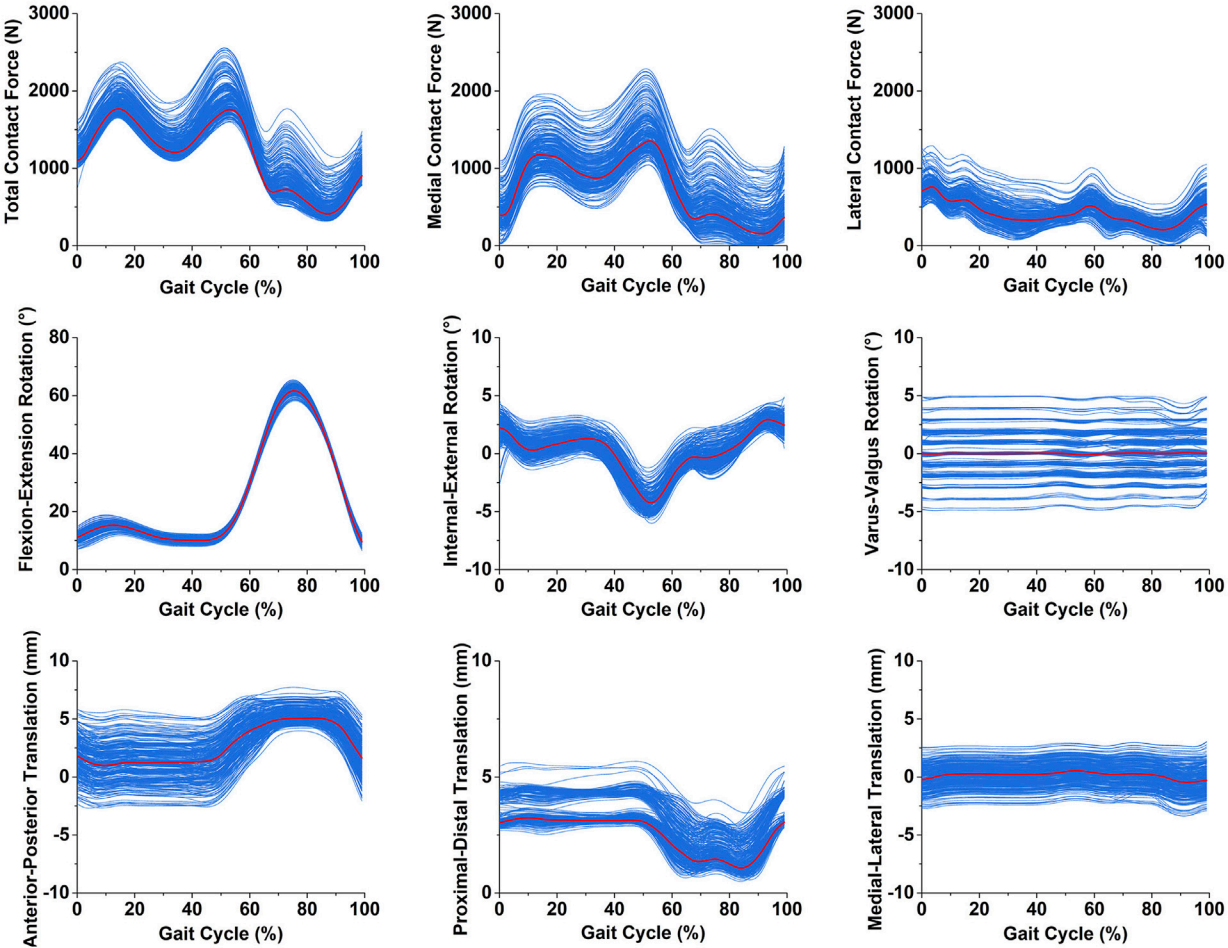
The knee biomechanics under different tibial component malrotation during a walking gait after total knee arthroplasty (the red line represent knee biomechanics under tibial component neutral position during a walking gait; the blue line represent knee biomechanics under different tibial component malrotation during a walking gait).

The knee total contact forces, medial contact forces, lateral contact forces, flexion-extension rotation, internal-external rotation, varus-valgus rotation, anterior-posterior translation, proximal-distal translation, and medial-lateral translation during a walking gait under different tibial component malrotation in the range of ±5° in the three directions were presented in Figure 3.

The comparison of ground truth values and deep learning prediction for knee contact forces under different tibial component malrotation during walking gait after TKA was presented in Table 2. For the training set, the RMSE for total contact force, medial contact force, and lateral contact force were 7.77, 33.90, and 11.17 N, respectively, as predicted by the deep learning model. The Pearson correlation coefficient for total contact force, medial contact force, and lateral contact force were 0.999, 0.995, and 0.999, respectively, as predicted by the deep learning model. For the validation set, the RMSE for total contact force, medial contact force, and lateral contact force were 32.54, 37.41, and 15.47 N, respectively, as predicted by the deep learning model. The Pearson correlation coefficient for total contact force, medial contact force, and lateral contact force were 0.995, 0.995, and 0.999, respectively, as predicted by the deep learning model. For the test set, the RMSE for total contact force, medial contact force, and lateral contact force were 38.44, 51.78, and 19.74 N, respectively, as predicted by the deep learning model. The Pearson correlation coefficient for total contact force, medial contact force, and lateral contact force were 0.995, 0.993, and 0.999, respectively, as predicted by the deep learning model.

**TABLE 2:** The comparison of ground truth values and deep learning prediction for knee contact forces under different tibial component malrotation during a walking gait after total knee arthroplasty.

	Training set		Validation set		Test set	
Predicted Feature	RMSE (N)	*ρ*	RMSE (N)	*ρ*	RMSE (N)	*ρ*
Total contact forces	7.77	0.999	32.54	0.995	38.44	0.995
Medial contact forces	33.90	0.995	37.41	0.995	51.78	0.993
Lateral contact forces	11.68	0.999	15.47	0.999	19.74	0.999

The comparison of ground truth values and four machine learning predictions for knee contact forces under different tibial component malrotation during a walking gait after TKA was presented in Table 3. For the Random Forest regression model (test set), the RMSE and Pearson correlation coefficient for total contact force, medial contact force, and lateral contact force were 63.15, 58.51, and 23.93 N, respectively, and 0.987, 0.992, and 0.998, respectively. For the AdaBoost regression model (test set), the RMSE and Pearson correlation coefficient for total contact force, medial contact force, and lateral contact force were 75.15, 81.94, and 34.84 N, respectively, and 0.983, 0.982, and 0.994, respectively. For the Gradient Boosting regression model (test set), the RMSE and Pearson correlation coefficient for total contact force, medial contact force, and lateral contact force were 65.98, 58.33, and 30.22 N, respectively, and 0.986, 0.992, and 0.994, respectively. For the Voting regression model (test set), the RMSE and Pearson correlation coefficient for total contact force, medial contact force, and lateral contact force were 62.32, 70.55, and 24.85 N, respectively, and 0.987, 0.984, and 0.998, respectively.

**TABLE 3:** The comparison of ground truth values and machine learning prediction for knee contact forces under different tibial component malrotation during a walking gait after total knee arthroplasty.

	Total contact forces		Medial contact forces		Lateral contact forces	
Regression Models	RMSE(N)	*ρ*	RMSE(N)	*ρ*	RMSE(N)	*ρ*
Random Forest	63.15	0.987	58.51	0.992	23.93	0.998
AdaBoost	75.15	0.983	81.94	0.982	34.84	0.994
Gradient Boosting	65.98	0.986	58.33	0.992	30.22	0.994
Voting	62.32	0.987	70.55	0.984	24.85	0.998

The comparison of ground truth values and deep learning prediction for knee kinematics under different tibial component malrotation during a walking gait after TKA was presented in Table 4. For the training set, the RMSE for flexion-extension rotation, internal-external rotation, varus-valgus rotation, anterior-posterior translation, proximal-distal translation, and medial-lateral translation were 0.07°, 0.04°, 0.11°, 0.04 mm, 0.03, and 0.05 mm, respectively, as predicted by the deep learning model. The Pearson correlation coefficient for flexion-extension rotation, internal-external rotation, varus-valgus rotation, anterior-posterior translation, proximal-distal translation, and medial-lateral translation were 0.999, 0.999, 0.998, 0.999, 0.999, and 0.998, respectively, as predicted by the deep learning model. For the validation set, the RMSE for flexion-extension rotation, internal-external rotation, varus-valgus rotation, anterior-posterior translation, proximal-distal translation, and medial-lateral translation were 0.12°, 0.20°, 0.10°, 0.20, 0.29, and 0.07 mm, respectively, as predicted by the deep learning model. The Pearson correlation coefficient for flexion-extension rotation, internal-external rotation, varus-valgus rotation, anterior-posterior translation, proximal-distal translation, and medial-lateral translation were 0.998, 0.996, 0.998, 0.997, 0.997, and 0.999, respectively, as predicted by the deep learning model. For the test set, the RMSE for flexion-extension rotation, internal-external rotation, varus-valgus rotation, anterior-posterior translation, proximal-distal translation, and medial-lateral translation were 0.19°, 0.18°, 0.11°, 0.16, 0.35, and 0.06 mm, respectively, as predicted by the deep learning model. The Pearson correlation coefficient for flexion-extension rotation, internal-external rotation, varus-valgus rotation, anterior-posterior translation, proximal-distal translation, and medial-lateral translation were 0.998, 0.998, 0.998, 0.998, 0.997, and 0.999, respectively, as predicted by the deep learning model.

**TABLE 4:** The comparison of ground truth values and deep learning prediction for knee kinematics under different tibial component malrotation during a walking gait after total knee arthroplasty.

	Training set		Validation set		Test set	
Predicted Features	RMSE	*ρ*	RMSE	*ρ*	RMSE	*ρ*
Flexion-Extension Rotation (°)	0.07	0.999	0.12	0.998	0.19	0.998
Internal-External Rotation (°)	0.04	0.999	0.20	0.996	0.18	0.998
Varus-Valgus Rotation (°)	0.11	0.998	0.10	0.998	0.11	0.998
Anterior-Posterior Translation (mm)	0.04	0.999	0.20	0.997	0.16	0.998
Proximal-Distal Translation (mm)	0.03	0.999	0.29	0.997	0.35	0.997
Medial-Lateral Translation (mm)	0.05	0.998	0.07	0.999	0.06	0.999

The comparison of ground truth values and machine learning prediction for knee kinematics (rotation) under different tibial component malrotation during a walking gait after TKA was presented in Table 5. For the Random Forest regression model (test set), the RMSE and Pearson correlation coefficient for flexion-extension rotation, internal-external rotation, and varus-valgus rotation were 0.13°, 0.28°, and 0.22°, respectively, and 0.999, 0.996, and 0.996, respectively. For the AdaBoost regression model (test set), the RMSE and Pearson correlation coefficient for flexion-extension rotation, internal-external rotation, and varus-valgus rotation were 0.23°, 0.29°, and 0.24°, respectively, and 0.998, 0.995, and 0.996, respectively. For the Gradient Boosting regression model (test set), the RMSE and Pearson correlation coefficient for flexion-extension rotation, internal-external rotation, and varus-valgus rotation were 0.14°, 0.25°, and 0.25°, respectively, and 0.999, 0.996, and 0.996, respectively. For the Voting regression model (test set), the RMSE and Pearson correlation coefficient for flexion-extension rotation, internal-external rotation, and varus-valgus rotation were 0.16°, 0.31°, and 0.24°, respectively, and 0.999, 0.995, and 0.996, respectively.

**TABLE 5:** The comparison of ground truth values and machine learning prediction for knee kinematics (rotation) under different tibial component malrotation during a walking gait after total knee arthroplasty.

	Flexion-extension rotation (°)		Internal-external rotation (°)		Varus-valgus rotation (°)	
Regression Models	RMSE	ρ	RMSE	ρ	RMSE	ρ
Random Forest	0.13	0.999	0.28	0.996	0.22	0.996
AdaBoost	0.23	0.998	0.29	0.995	0.24	0.996
Gradient Boosting	0.14	0.999	0.25	0.996	0.25	0.996
Voting	0.16	0.999	0.31	0.995	0.24	0.996

The comparison of ground truth values and machine learning prediction for knee kinematics (translation) under different tibial component malrotation during a walking gait after TKA was presented in Table 6. For the Random Forest regression model (test set), the RMSE and Pearson correlation coefficient for anterior-posterior translation, proximal-distal translation, and medial-lateral translation were 0.30, 0.29, and 0.15 mm, respectively, and 0.995, 0.995, and 0.998, respectively. For the AdaBoost regression model (test set), the RMSE and Pearson correlation coefficient for anterior-posterior translation, proximal-distal translation, and medial-lateral translation were 0.31 mm, 0.47, and 0.22 mm, respectively, and 0.995, 0.994, and 0.997, respectively. For the Gradient Boosting regression model (test set), the RMSE and Pearson correlation coefficient for anterior-posterior translation, proximal-distal translation, and medial-lateral translation were 0.29, 0.56, and 0.18 mm, respectively, and 0.996, 0.992, and 0.998, respectively. For the Voting regression model (test set), the RMSE and Pearson correlation coefficient for anterior-posterior translation, proximal-distal translation, and medial-lateral translation were 0.34, 0.60, and 0.15 mm, respectively, and 0.995, 0.992, and 0.998, respectively.

**TABLE 6:** The comparison of ground truth values and machine learning prediction for knee kinematics (translation) under different tibial component malrotation during a walking gait after total knee arthroplasty.

	Anterior-posterior translation (mm)		Proximal-distal translation (mm)		Medial-lateral translation (mm)	
Regression Models	RMSE	ρ	RMSE	ρ	RMSE	ρ
Random Forest	0.30	0.995	0.29	0.995	0.15	0.998
AdaBoost	0.31	0.995	0.47	0.994	0.22	0.997
Gradient Boosting	0.29	0.996	0.56	0.992	0.18	0.998
Voting	0.34	0.995	0.60	0.992	0.15	0.998

## 4 Discussion

The most important findings of the present study were that the deep learning method were capable of accurately and reliability predicting knee contact forces (total contact force, medical contact force, and lateral contact force) and kinematics (flexion-extension rotation, internal-external rotation, varus-valgus rotation, anterior-posterior translation, proximal-distal translation, and medial-lateral translation) with different tibial component malrotation during a walking gait after TKA, compared to four conventional machine learning methods.

The dataset of knee contact forces and kinematics with different tibial component malrotation during a walking gait after TKA (Figure 3) was based on our developed and validated musculoskeletal multibody dynamics model (Chen et al., 2015; Chen et al., 2016; Zhang et al., 2023). Our previous studies have shown that the musculoskeletal multibody dynamics model could accurately predict knee contact forces and kinematics during a walking gait after TKA (Zhang et al., 2019; Zhang et al., 2020), which were generally in excellent agreement with the experimental data previously measured in the patients via instrumented prosthesis. Nonetheless, the musculoskeletal multibody dynamics model is subject to time-intensive procedures and limited in its capacity to promptly generate the biomechanical response of the patient’s knee joint after TKA. During the preoperative planning phase of a patient’s knee arthroplasty, the musculoskeletal multibody dynamics model may not be able to quickly offer the surgeon precise information regarding the biomechanical relationship between the prosthesis position and the knee biomechanics in TKA because of the time-consuming of computational models. The deep/machine learning models that were developed in this paper to predict the knee contact forces and kinematics with different tibial component malrotation during a walking gait after TKA effectively overcomes the limitations of musculoskeletal multi-body dynamics models in clinical applications. These models could quickly and accurately predict knee biomechanics for different component malrotation to facilitate optimal preoperative planning and intraoperative guidance for TKA procedures.

The RMSE (38.44 N) (testing set) of the deep learning method in predicting the total contact force under different tibial component malrotation during a walking gait after TKA was significantly lower than the RMSE (62.32–75.15 N) (testing set) of four machine learning methods in predicting the total contact force under different tibial component malrotation after TKA. Similarly, the RMSE (51.78 N) (testing set) of the deep learning method in predicting the medial contact force under different tibial component malrotation during a walking gait after TKA was lower than the RMSE (58.33–81.94 N) (testing set) of four machine learning methods in predicting the medial contact force under different tibial component malrotation after TKA. The RMSE (19.74 N) (testing set) of the deep learning method in predicting the lateral contact force under different tibial component malrotation during a walking gait after TKA was lower than the RMSE (23.93–34.84 N) (testing set) of four machine learning methods in predicting the lateral contact force under different tibial component malrotation after TKA. These results revealed that compared to four conventional machine learning methods, the developed deep learning method had higher accuracy in predicting total contact force, medial contact force, and lateral contact force under different tibial component malrotation during a walking gait after TKA, as evidenced by the relatively low RMSE values and high Pearson correlation coefficients (Tables 2, 3).

Furthermore, the RMSE (0.18°) (testing set) of the deep learning method in predicting the internal-external rotation under different tibial component malrotation during a walking gait after TKA was significantly lower than the RMSE (0.25°–0.31°) (testing set) of four machine learning methods in predicting the internal-external rotation under different tibial component malrotation after TKA. Similarly, the RMSE (0.11°) (testing set) of the deep learning method in predicting the varus-valgus rotation under different tibial component malrotation during a walking gait after TKA was significantly lower than the RMSE (0.22°–0.25°) (testing set) of four machine learning methods in predicting the varus-valgus rotation under different tibial component malrotation after TKA. The RMSE (0.16 mm) (testing set) of the deep learning method in predicting the anterior-posterior translation under different tibial component malrotation during a walking gait after TKA was significantly lower than the RMSE (0.29–0.34 mm) (testing set) of four machine learning methods in predicting the anterior-posterior translation under different tibial component malrotation after TKA. The RMSE (0.06 mm) (testing set) of the deep learning method in predicting the medial-lateral translation under different tibial component malrotation during a walking gait after TKA was significantly lower than the RMSE (0.15–0.22 mm) (testing set) of four machine learning methods in predicting the medial-lateral translation under different tibial component malrotation after TKA. These results indicated that compared to four machine learning methods, the developed deep learning model had higher accuracy in predicting internal-external rotation, varus-valgus rotation, anterior-posterior translation, and medial-lateral translation under different tibial component malrotation during a walking gait after TKA, as evidenced by the relatively low RMSE values and high Pearson correlation coefficients (Tables 4–6).

However, the RMSE (0.19°) (testing set) of the deep learning method in predicting the flexion-extension rotation under different tibial component malrotation during a walking gait after TKA (Table 4) was marginally higher than the RMSE (testing set) of the Random Forest regression model (0.13°), Gradient Boosting regression model (0.14°), and Voting regression model (0.16°), but was lower than the AdaBoost regression model (0.23°) in predicting the flexion-extension rotation under different tibial component malrotation after TKA (Table 5). The RMSE (0.35 mm) (testing set) of the deep learning method in predicting the proximal-distal translation under different tibial component malrotation during a walking gait after TKA (Table 4) was marginally higher than the RMSE (testing set) of the random forest regression model (0.29 mm), but was still lower than the RMSE (testing set) of AdaBoost regression model (0.47 mm), Gradient Boosting regression model (0.56 mm), and Voting regression model (0.60 mm) in predicting the proximal-distal translation under different tibial component malrotation after TKA (Table 6). Nevertheless, the deep learning model demonstrated higher accuracy in predicting the flexion-extension rotation and the proximal-distal translation under different tibial component malrotation during a walking gait after TKA, compared to other machine learning methods.

Several limitations of this study should be discussed. Firstly, the dataset of knee contact forces and kinematics with different tibial component malrotation during a walking gait after TKA was established using our developed musculoskeletal multibody dynamics model based on a patient’s experimental data. The objective of this study was to develop deep/machine learning methods to predict knee biomechanics for different tibial component malrotation during a walking gait in TKA. The focus was on establishing the relationship between different tibial component malrotation and the knee biomechanics specifically within TKA. The main intention of this study was not to predict knee biomechanics from different gaits across patients. Therefore, this study conclusively demonstrated that the deep learning method was able to predict the knee contact forces and kinematics accurately and quickly under different tibial component malrotation in TKA, which provides surgeons and surgical robots with a calibration safety zone for the preoperative planning and intraoperative guidance in TKA. Because of the limited number of patients, it was recommended that the reader construes the current study as a case series. This could potentially serve as a significant initial step for forthcoming extensive investigations, especially in large-scale research studies leveraging computer vision, deep learning and musculoskeletal simulation. The effect of different patients and prosthesis designs on knee biomechanics for different component malrotation will continue to be explored in future work. Secondly, the use of mechanical alignment, anatomical alignment, and kinematic alignment in TKA remains controversial. The alignment of the prosthesis in this study was based on the principle of mechanical alignment, and the biomechanical effects of different tibiofemoral component malrotation under different alignment principles will also be further investigated in future work. Thirdly, the objective of this study was to utilize the developed deep/machine learning methods to predict knee biomechanics during a walking gait in TKA for different tibial component malrotation. However, it is important to note that the biomechanical effects of different femoral component malrotation should be investigated in future work. Fourthly, walking gait was taken into consideration in this study since it is the activity that occurs the most frequently in day-to-day life, and direct *in vivo* measurements of joint contact forces derived from instrumented TKA prostheses are available for use in model validation. Deep learning models of knee biomechanics under different tibiofemoral component malrotation after TKA with various gait patterns, such as squatting, stair climbing, and jumping, will be investigated comprehensively in future work. Finally, in this study, four conventional machine learning methods and a deep learning method were employed to predict knee contact forces and kinematics with different tibial component malrotation during a walking gait in TKA. A larger dataset should be involved in future studies. Additional investigation is necessary to explore the optimization of both the sample size and algorithm.

## 5 Conclusion

The deep learning method developed in the present study was able to accurately and rapidly predict knee contact forces and kinematics with different tibial component malrotation during a walking gait after TKA, outperforming four conventional machine learning methods. The proposed method provided surgeons and surgical robots with the capability to establish a calibration safety zone, a critical aspect in ensuring precise alignment for both preoperative surgical planning and intraoperative robotic-assisted surgical navigation.

## Data Availability

The raw data supporting the conclusion of this article will be made available by the authors, without undue reservation.
