# Supplementary material for: Prediction of knee biomechanics with different tibial component malrotations after total knee arthroplasty: conventional machine learning vs. deep learning
Source: Front Bioeng Biotechnol. 2024 Jan 8;11:1255625. doi: 10.3389/fbioe.2023.1255625 (PMC10800660; doi:10.3389/fbioe.2023.1255625)
Supplement: Supplementary file 1 [file Table1.docx]

Supplementary Table 1 The comparison of ground truth values and machine learning prediction for knee contact forces under different tibial component malrotation during a walking gait after total knee arthroplasty

|  | Training set | | | | | |
| --- | --- | --- | --- | --- | --- | --- |
| Regression Models | Total contact forces | | Medial contact forces | | Lateral contact forces | |
|  | RMSE(N) | *ρ* | RMSE(N) | *ρ* | RMSE(N) | *ρ* |
| Random Forest | 37.69 | 0.996 | 38.26 | 0.996 | 17.66 | 0.999 |
| AdaBoost | 46.18 | 0.992 | 54.08 | 0.990 | 25.30 | 0.998 |
| Gradient Boosting | 47.36 | 0.992 | 41.77 | 0.996 | 20.07 | 0.998 |
| Voting | 42.98 | 0.996 | 50.63 | 0.990 | 18.94 | 0.999 |
|  | Validation set | | | | | |
| Regression Models | Total contact forces | | Medial contact forces | | Lateral contact forces | |
|  | RMSE(N) | *ρ* | RMSE(N) | *ρ* | RMSE(N) | *ρ* |
| Random Forest | 50.47 | 0.990 | 42.86 | 0.996 | 20.52 | 0.998 |
| AdaBoost | 59.21 | 0.987 | 67.19 | 0.984 | 29.68 | 0.998 |
| Gradient Boosting | 54.76 | 0.990 | 46.38 | 0.994 | 26.81 | 0.994 |
| Voting | 50.63 | 0.990 | 59.02 | 0.987 | 21.37 | 0.998 |
