# Supplementary material for: Prediction of knee biomechanics with different tibial component malrotations after total knee arthroplasty: conventional machine learning vs. deep learning
Source: Front Bioeng Biotechnol. 2024 Jan 8;11:1255625. doi: 10.3389/fbioe.2023.1255625 (PMC10800660; doi:10.3389/fbioe.2023.1255625)
Supplement: Supplementary file 2 [file Table2.docx]

Supplementary Table 2 The comparison of ground truth values and machine learning prediction for knee kinematics (rotation) under different tibial component malrotation during a walking gait after total knee arthroplasty

|  | Training set | | | | | |
| --- | --- | --- | --- | --- | --- | --- |
| Regression Models | Flexion-Extension Rotation (°) | | Internal-External Rotation (°) | | Varus-Valgus Rotation (°) | |
|  | RMSE | ρ | RMSE | ρ | RMSE | ρ |
| Random Forest | 0.06 | 0.999 | 0.16 | 0.999 | 0.18 | 0.999 |
| AdaBoost | 0.15 | 0.999 | 0.17 | 0.999 | 0.21 | 0.998 |
| Gradient Boosting | 0.07 | 0.999 | 0.16 | 0.999 | 0.22 | 0.998 |
| Voting | 0.08 | 0.999 | 0.19 | 0.999 | 0.20 | 0.999 |
|  | Validation set | | | | | |
| Regression Models | Flexion-Extension Rotation (°) | | Internal-External Rotation (°) | | Varus-Valgus Rotation (°) | |
|  | RMSE | ρ | RMSE | ρ | RMSE | ρ |
| Random Forest | 0.10 | 0.999 | 0.26 | 0.998 | 0.20 | 0.998 |
| AdaBoost | 0.19 | 0.999 | 0.26 | 0.998 | 0.24 | 0.998 |
| Gradient Boosting | 0.11 | 0.999 | 0.22 | 0.999 | 0.24 | 0.998 |
| Voting | 0.12 | 0.999 | 0.27 | 0.998 | 0.22 | 0.998 |
