# Supplementary material for: Prediction of knee biomechanics with different tibial component malrotations after total knee arthroplasty: conventional machine learning vs. deep learning
Source: Front Bioeng Biotechnol. 2024 Jan 8;11:1255625. doi: 10.3389/fbioe.2023.1255625 (PMC10800660; doi:10.3389/fbioe.2023.1255625)
Supplement: Supplementary file 3 [file Table3.docx]

Supplementary Table 3 The comparison of ground truth values and machine learning prediction for knee kinematics (translation) under different tibial component malrotation during a walking gait after total knee arthroplasty

|  | Training set | | | | | |
| --- | --- | --- | --- | --- | --- | --- |
| Regression Models | Anterior-Posterior Translation (mm) | | Proximal-Distal Translation (mm) | | Medial-Lateral Translation (mm) | |
|  | RMSE | ρ | RMSE | ρ | RMSE | ρ |
| Random Forest | 0.23 | 0.998 | 0.20 | 0.999 | 0.12 | 0.999 |
| AdaBoost | 0.26 | 0.998 | 0.29 | 0.998 | 0.14 | 0.999 |
| Gradient Boosting | 0.20 | 0.999 | 0.35 | 0.998 | 0.11 | 0.999 |
| Voting | 0.25 | 0.998 | 0.39 | 0.995 | 0.12 | 0.999 |
|  | Validation set | | | | | |
| Regression Models | Anterior-Posterior Translation (mm) | | Proximal-Distal Translation (mm) | | Medial-Lateral Translation (mm) | |
|  | RMSE | ρ | RMSE | ρ | RMSE | ρ |
| Random Forest | 0.28 | 0.996 | 0.25 | 0.998 | 0.14 | 0.999 |
| AdaBoost | 0.30 | 0.996 | 0.38 | 0.995 | 0.18 | 0.998 |
| Gradient Boosting | 0.26 | 0.996 | 0.46 | 0.994 | 0.15 | 0.998 |
| Voting | 0.30 | 0.996 | 0.52 | 0.992 | 0.16 | 0.998 |
